# Phenotypic Complexity and Diagnostic Utility of Exome Sequencing in ASD: Real-World Insights from Colombia

**DOI:** 10.3390/ijms27198902

**Published:** 2026-10-07

**Authors:** Diego Alejandro Rodríguez Gutiérrez, Ronald Cárdenas Prieto, Paula Rueda-Gaitán, Natalia Gomez-Lopera, Carlos Estrada-Serrato, Mario Isaza-Ruget, Juan Javier López Rivera

**Affiliations:** 1 Laboratorio Clínico y de Patología, Clínica Colsanitas, Bogotá 111321, Colombiaronald.cardenas@colsanitas.com (R.C.P.); pauarueda@colsanitas.com (P.R.-G.); ext.natalia.gomezl@keralty.com (N.G.-L.);; 2 Fundación Universitaria Sanitas, Bogotá 110141, Colombia

**Keywords:** autism, NGS, HPO, Latin America

## Abstract

Autism spectrum disorder (ASD) is a genetically heterogeneous condition; however, genomic data from Latin American populations remain limited. We investigated the molecular and phenotypic landscape of ASD in 916 Colombian patients who underwent whole-exome sequencing (WES) between 2022 and 2025. Clinical and genomic data were retrospectively analyzed, and phenotypes were standardized using Human Phenotype Ontology (HPO) terms. Variants were classified per ACMG/ClinGen guidelines, and we assessed phenotypic burden, predictors of molecular diagnosis, copy number variants (CNVs), and functional convergence of candidate genes. A molecular diagnosis was identified in 112 patients (12.2%), including 82 with variants in ASD-associated genes or regions and 30 with findings related to other clinical features. We identified 16 novel pathogenic/likely pathogenic variants, along with 10 CNVs. Among trio-based patients, 70.9% of molecular diagnoses were de novo. Higher phenotypic burden was associated with molecular diagnosis, and global developmental delay was the strongest predictor after correction for multiple testing. Functional enrichment analysis of candidate genes showed convergence on pathways related to neuronal development, synaptic function, and ion transport. These findings highlight the genetic and phenotypic heterogeneity of ASD and support the value of integrating standardized genomic and phenotypic characterization in Colombian populations.

## 1. Introduction

Autism spectrum disorder (ASD) is a heterogeneous neurodevelopmental condition defined by impairments in social communication and interaction, as well as restrictive and repetitive behaviors, according to current diagnostic criteria (DSM-5) [1]. ASD is frequently associated with comorbidities, including intellectual disability (ID), epilepsy, sleep disturbances, gastrointestinal disturbances, and difficulties with attention and emotional self-regulation. Neuropsychiatric disorders such as attention-deficit/hyperactivity disorder (ADHD), anxiety, behavioral problems, depression, and developmental delays are also commonly observed. Although ASD can be linked to genetic syndromes such as fragile X syndrome, neurofibromatosis, and tuberous sclerosis, it most often presents as a nonsyndromic condition [2,3]. The prevalence and nature of comorbidities depend on clinical characteristics. For example, epilepsy affects 6% to 30% of individuals with ASD, with higher rates, from 21.5% to 70%, in those with intellectual disabilities (IQ < 70). The increased prevalence of certain health conditions in individuals with ASD suggests a possible shared etiology between these comorbidities and the core features of ASD [4]. This broad clinical variability indicates that ASD represents a spectrum of neurodevelopmental presentations with diverse genetic and phenotypic architectures, rather than a single biological entity.

Although the etiology of ASD is not fully understood, genetic factors are recognized as central contributors. Twin and family studies exhibit high heritability, with concordance rates ranging from 26% to 96% in monozygotic twins and from 0% to 30% in dizygotic twins. The recurrence risk in first-degree relatives is approximately 75%higher than in the general population [2,5,6]. However, the specific genetic mechanisms underlying ASD remain incompletely elucidated. Current evidence supports a complex genetic architecture, with most risk attributed to common variants that exert small additive effects across the genome [7,8]. High-impact de novo variants (DNVs) and rare copy-number variants (CNVs) account for approximately 10% to 20% of cases. These rare, high-penetrance variants are more frequently identified in patients with comorbidities such as intellectual disability (ID) and developmental disorders (DD) [9].

More than 1000 genes are estimated to contribute to ASD, mainly through pathways involved in cellular and neuronal development, synaptic plasticity regulation, and cellular motility [10]. However, most available data are derived from studies of individuals of European descent, resulting in a significant knowledge gap regarding other populations. Understudied groups, such as the Colombian population, may afford valuable insights into novel genes and pathways linked to ASD pathogenesis. The Colombian population, characterized by a tri-hybrid genetic composition (European, African, and Amerindian) [11], offers an opportunity to investigate genetic variants that may be underrepresented in global databases. Distinct genomic ancestral backgrounds can reveal disease-modifying variants and specific risk loci.

Whole exome sequencing (WES) is a key tool for identifying genetic variants associated with ASD. This approach permits the analysis of mutations in known genes and the discovery of novel genes, consequently expanding genetic databases and increasing diagnostic accuracy. In Colombia, genetic research on ASD remains limited. The “Genetic Heterogeneity of Autism” project, initiated in 2011 by the Center for Genetic Research on Human Diseases (CIGEN) at Universidad de los Andes, in collaboration with the Colombian Autism League and other institutions, has identified specific genetic variants in this population. For example, exome analysis of four Colombian trios revealed two novel missense single-nucleotide variants (SNVs) in the same patient: *ALDH1A3* (c.1514T>C, p.I505T) and *FOXN1* (c.146C>T, p.S49L) [12,13]. However, the small sample size precluded a thorough characterization of the genetic profile of ASD in Colombian patients.

Dealing with these limitations requires precise and standardized phenotypic characterization to fully leverage clinical genomics. The Human Phenotype Ontology (HPO) provides a standardized structure for capturing clinical features in a computable format and is increasingly important in genomic medicine. HPO-based phenotyping facilitates genotype–phenotype correlation, enhances variant prioritization, and facilitates systematic comparison of clinical features among patients and cohorts. In ASD, where phenotypic heterogeneity is considerable, integrating HPO terms with genomic data may help identify clinical predictors of molecular diagnosis and reveal biologically meaningful subgroups. This study analyzed WES data from 916 Colombian patients with clinical features of ASD. By combining molecular findings with curated HPO-based phenotypic data, the objectives were to characterize the positivity rate and molecular spectrum of pathogenic and likely pathogenic variants, evaluate phenotypic predictors of a positive molecular diagnosis, assess the contribution of copy-number variants, and explore the functional convergence of candidate genes not previously established as ASD-associated.

## 2. Results

### 2.1. Study Cohort

From 2022 to 2025, a total of 8346 patients underwent evaluation using single, duo, or trio whole-exome sequencing (WES). Based on clinical histories, phenotype-based filtering was applied to identify a subcohort of 916 patients presenting with clinical features of autism spectrum disorder (ASD). Within this specific cohort, sequencing was distributed among singletons (n = 295), duos (n = 25), and trios (n = 596). The mean age of these patients was 7 years (IQR = 4), comprising 200 females (21.8%) and 716 males (78.2%).

We identified 112 patients with positive results, corresponding to a diagnostic rate of 12.2%. Among them, 82 had findings in genes or regions with a confirmed association with autism, and 30 positive results were associated with other characteristics of the patient’s clinical picture. Within the isolated ASD subgroup (9.8%, n = 90), the pathogenic/likely pathogenic (P/LP) positivity rate was 3%, while variants of uncertain significance (VUS) accounted for 21%. The low P/LP positivity rate in isolated ASD suggests that WES-detectable coding variants explain only a small proportion of cases, while negative WES results do not exclude an underlying genetic etiology.

### 2.2. Molecular Spectrum of Pathogenic and Likely Pathogenic SNVs

A total of 73 pathogenic or likely pathogenic (P/LP) small variants were identified across 54 distinct genes. Characterization of the molecular spectrum revealed that missense variants were the most prevalent (35.6%, n = 26), followed by frameshift (27.4%, n = 20) and nonsense (23.3%, n = 17) alterations, alongside six splice-site variants (8.2%), 2 single-nucleotide deletions, and a single intronic variant. Beyond single-nucleotide changes, ten CNVs disrupting critical ASD-related loci were resolved. Remarkably, within the trio-sequenced subset (n = 62), the phenotypic driver was confirmed as a de novo mutation in 70.9% of cases (n = 44), underscoring a dominant genetic architecture. Comprehensive annotation and literature curation are detailed in Table 1, with previously unreported variants designated by an asterisk (*).

Among the pathogenic and likely pathogenic variants identified, several occurred in genes that are not currently recognized as definitive ASD-associated genes (Appendix A Table A1). These genes were primarily associated with neurodevelopmental disorders, epileptic *encephalopathies*, channelopathies, metabolic disorders, and multisystem syndromes. Notably, pathogenic or likely pathogenic variants were detected in genes such as *SCN9A*, *TUBB2A*, *MN1*, *LARS2*, and *SLC12A2*, for which direct evidence supporting a role in ASD remains limited or absent. Although these genes have been implicated in neurological or developmental disorders, they are not currently classified as established ASD genes in major ASD resources such as SFARI Gene. Several additional genes, including *DNM1*, *MAST3*, *MAPK8IP3*, and *ABCC8*, have been reported in developmental disorder databases or neurodevelopmental disease cohorts but have not been consistently associated with ASD.

**Figure A1:**
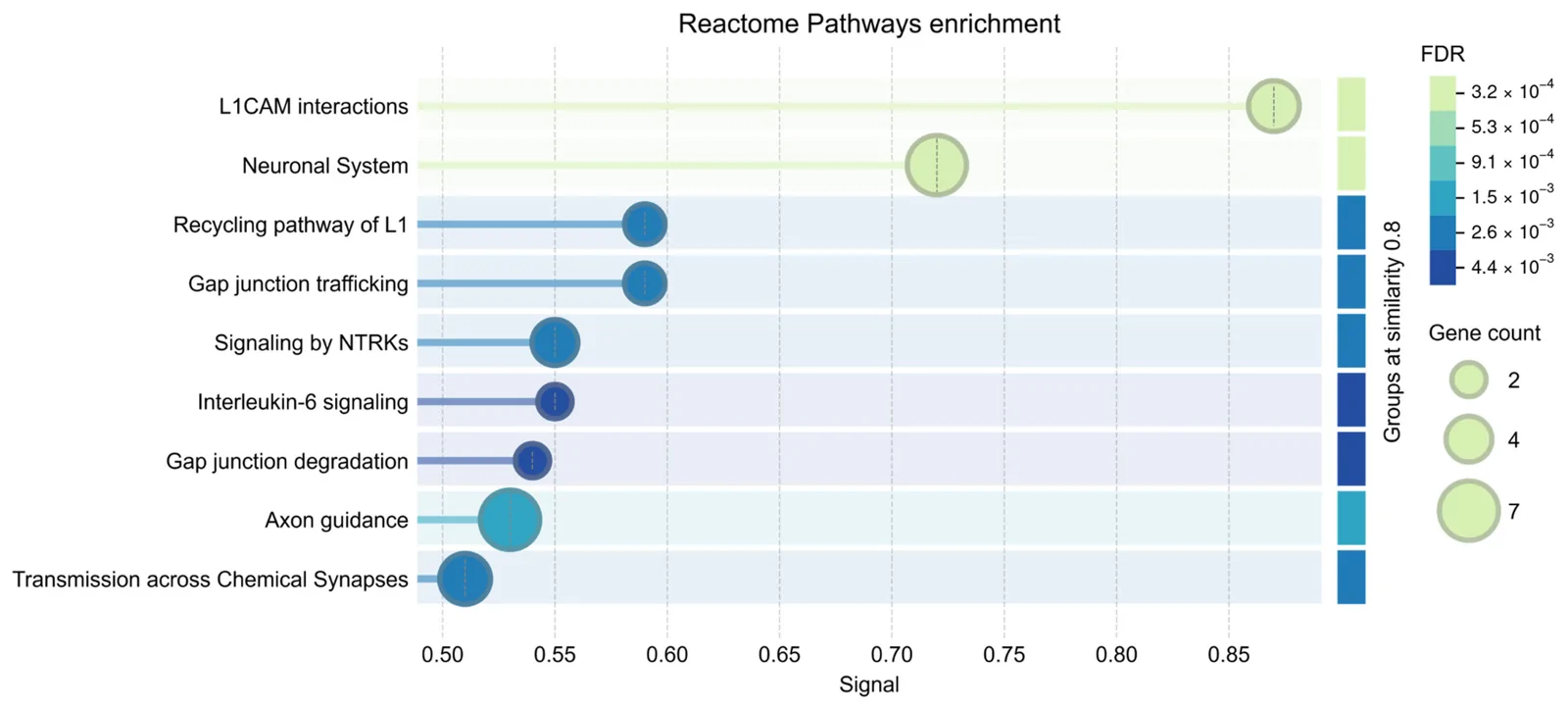
Pathway enrichment analysis of candidate genes identified in Colombian ASD patients.

To investigate the biological relevance of these candidate genes, functional enrichment analyses were performed using only genes without established ASD associations. Reactome pathway analysis revealed significant enrichment of pathways involved in neuronal development and synaptic function, including *Neuronal System*, *Transmission across Chemical Synapses*, *Axon Guidance*, *Signaling by NTRKs*, *and L1CAM Interactions* (Appendix A Figure A1). Additional enrichment was observed for pathways related to gap junction dynamics and intracellular trafficking.

Consistent with these findings, Gene Ontology analysis demonstrated enrichment of biological processes associated with ion transport, calcium transport, transmembrane transport, and regulation of ion homeostasis. Human Phenotype Ontology enrichment further identified neurological phenotypes such as epileptiform activity, generalized epileptiform EEG abnormalities, and hypsarrhythmia, as well as cardiovascular and developmental features including arrhythmias, abnormal heart valve morphology, dysphagia, and cubitus valgus.

### 2.3. CNV Landscape in ASD Diagnostic Evaluation

Diagnostic CNVs associated with autism were identified in 10 of the 916 patients analyzed (1.09%). Within the subgroup of 82 patients with confirmed genetic variants related to ASD, CNVs accounted for 12.9% (10/87) of molecular diagnoses, thereby highlighting the fundamental role of structural variants in the genetic architecture of the Colombian population with ASD.

Among the 10 diagnostic CNVs detected, microdeletions predominated, accounting for 80% (n = 8) of the structural alterations, while microduplications accounted for the remaining 20%. The genomic size of these variants exhibited a substantial range, from microdeletions of a few kilobases, exemplified by the 6.4-kilobase deletion, to substantial chromosomal segment imbalances extending up to 7.0 megabases at the 10q27.13-26.3 locus. The mean size calculated for the pathogenic or likely pathogenic CNVs detected was 2.94 megabases (Mb).

It is noteworthy that the structural variants directly affected loci and high-penetrance genes closely associated with neurodevelopmental disorders and recurrent syndromic genomic regions. The presence of microdeletions affecting the 22q13.33 locus was identified in three unrelated patients, directly affecting the SHANK3 gene, which is considered high risk for ASD. This finding was consistent with the clinical presentation of Phelan-McDermid syndrome. A comprehensive compendium of genomic coordinates (GRCh37/hg19) and copy number statuses for all 10 diagnostic structural variants is provided in Table 2.

### 2.4. Phenotypic Predictors of Molecular Diagnosis

#### 2.4.1. Phenotypic Burden and Positivity Rate

**Figure 1:**
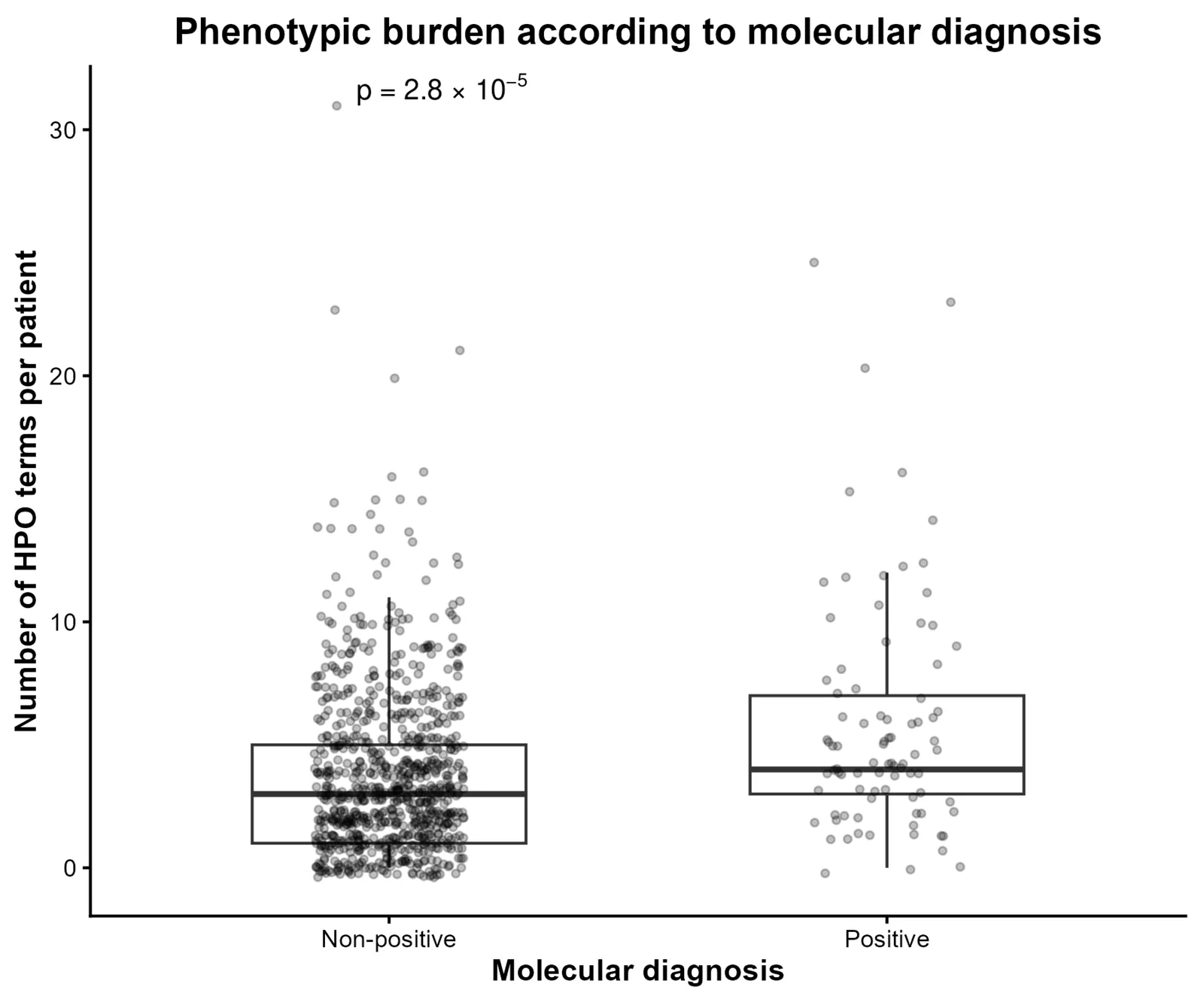
Phenotypic burden according to molecular diagnosis. Patients with a positive molecular diagnosis showed a significantly higher number of HPO terms per individual than non-positive patients [median 4.0 (IQR 3.0–7.0) vs. 3.0 (IQR 1.0–5.0), Wilcoxon rank-sum test, *p* < 0.001].

To investigate the relationship between clinical complexity and molecular positivity rate, we compared the number of Human Phenotype Ontology (HPO) terms between patients with and without a positive molecular diagnosis. Patients carrying pathogenic or likely pathogenic variants exhibited a significantly greater phenotypic burden than non-positive patients, with a median of 4.0 HPO terms per individual (IQR: 3.0–7.0) compared with 3.0 HPO terms (IQR: 1.0–5.0) among patients without a molecular diagnosis (*p* < 0.001) (Figure 1). These findings indicate that increased phenotypic complexity is associated with a higher probability of identifying a clinically relevant genetic alteration.

#### 2.4.2. Phenotypic Features Associated with a Positive Molecular Diagnosis

**Figure 2:**
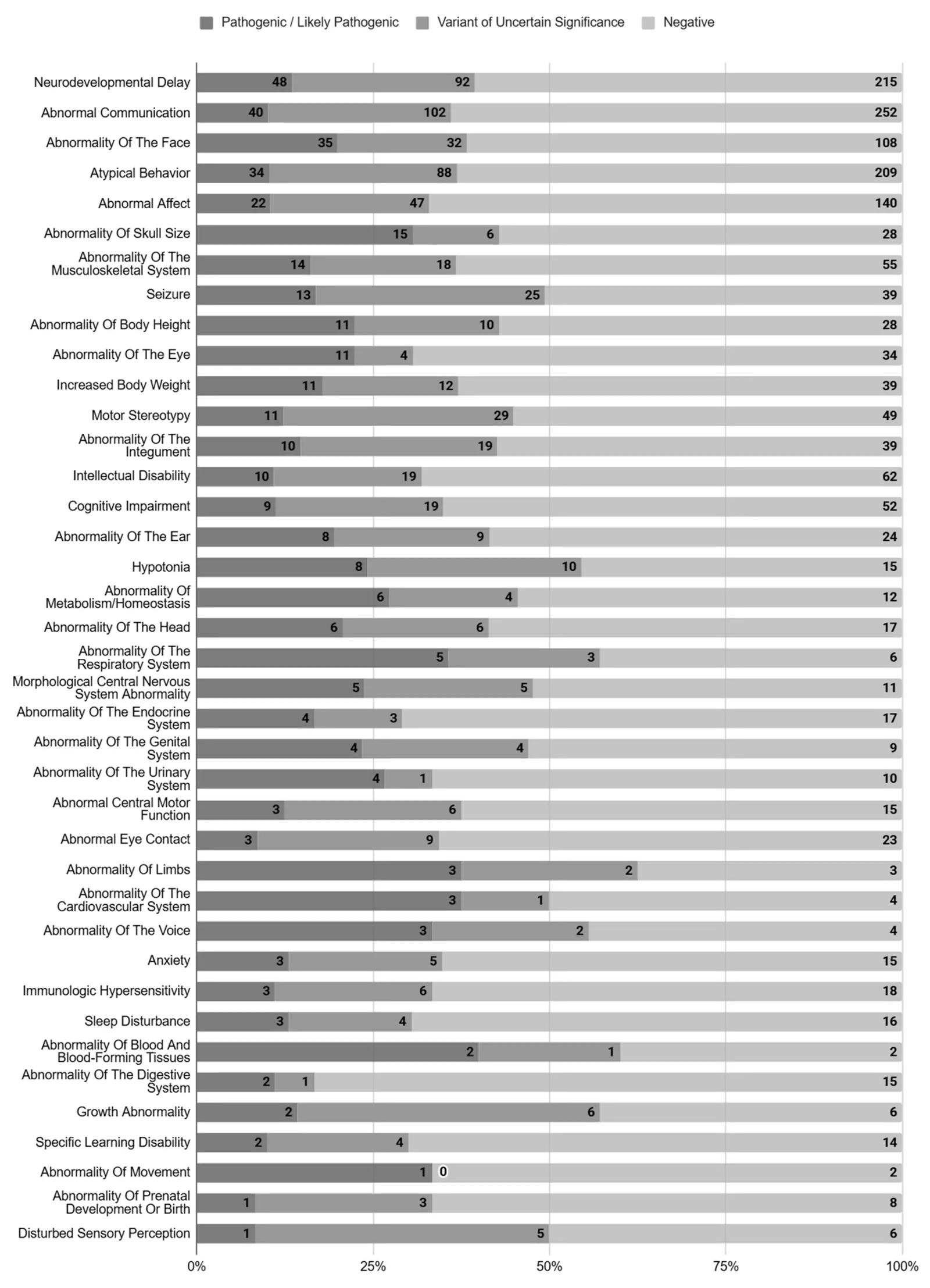
Distribution of HPO terms according to genetic results in our cohort. The distribution of the main HPO phenotypic terms is shown according to genetic results classified as pathogenic (P), likely pathogenic (LP), variants of uncertain significance (VUS), and negative. Values represent the number of patients presenting each phenotypic term within each genetic result category.

A total of 186 HPO terms were initially identified among patients with a positive molecular diagnosis. After semantic similarity analysis and clinical review, 39 clinically relevant phenotypic features were selected for association testing. Several phenotypic features were associated with an increased positivity rate (Appendix A Table A2). Among them, global developmental delay showed the strongest and most robust association with a positive molecular diagnosis, with a positivity rate of 20.7% and approximately three-fold higher odds of identifying a pathogenic or likely pathogenic variant compared with patients without this phenotype (OR = 3.07, 95% CI: 1.76–5.24, FD R-adjusted p = 0.034) (Figure 2).

Additional neurodevelopmental and syndromic features showed nominal associations with molecular diagnosis, including microcephaly (yield = 55.6%, OR = 12.5), neurodevelopmental abnormality (yield = 36.8%, OR = 5.94), macrocephaly (yield = 25.0%, OR = 3.45), short stature (yield = 28.6%, OR = 4.09), seizures (yield = 19.7%, OR = 2.57), abnormal facial shape (yield = 26.9%, OR = 3.72), and hypotonia (yield = 22.6%, OR = 2.93). However, none of these associations remained statistically significant after correction for multiple testing (Appendix A Table A2). Several rare phenotypes, including skeletal muscle atrophy, receptive language delay, abnormal affect, weight loss, neonatal asphyxia, and poor speech, demonstrated high diagnostic yields and large effect sizes. Nevertheless, these estimates were based on a limited number of observations and should therefore be interpreted cautiously.

## 3. Discussion

Autism spectrum disorder (ASD) is a neurodevelopmental condition with a strong genetic component, characterized by social communication deficits and repetitive behaviors [14]. The World Health Organization estimates that autism affects 0.76% of children globally [15], with higher rates (1.14%) observed in males (average male-female ratio of 4–5:1) [16]. In Colombia, ASD has emerged as an increasingly recognized public health issue. According to the study published by Colombia’s Ministry of Health and Social Protection, titled Analysis of the Mental Health Situation with an Emphasis on Social Determinants: Colombia, 2024, the prevalence rate of ASD in the country saw a marked increase between 2019 and 2023, rising from 30.3 to 71.9 cases per 100,000 inhabitants; higher prevalence was observed among males, with rates up to four times higher than those recorded among females [17].

As a leading center for genomics in Latin America, Colombia has made significant efforts over the past decade to implement state-of-the-art precision diagnostics for neurodevelopmental disorders. Thanks to improved access to healthcare, diagnosis rates for these disorders have risen, and patients now benefit from comprehensive care pathways. Specifically in Colombia, the national health system funds testing through Health Promoting Entities (EPS, by its acronym in Spanish), which has considerably narrowed the precision medicine gap. However, a lack of research on ASD in Colombia has hindered the understanding of the true disease burden at the national level.

This study presents a large cohort of patients with autism spectrum disorder (ASD), one of the largest recorded in a clinical diagnostic setting in Colombia and Latin America, thereby contributing to a better understanding of the disorder’s molecular basis. A total of 916 patients with clinical characteristics of ASD were analyzed; the median age was 7 years (interquartile range [IQR] = 4). Of these, 200 were female (21.8%), and 716 were male (78.2%), demonstrating a marked male predominance consistent with the global epidemiological profile of ASD. Among the total number of patients, 112 exhibited positive genetic outcomes, corresponding to a positivity rate of 12.2%. Variants in genes or regions previously associated with ASD were identified in 82 patients (73.2%), whereas variants associated with other clinical characteristics were detected in the remaining 30 patients (26.8%). These findings also reveal a notably low positivity rate in the subgroup with isolated ASD (3%) and a high rate of variants of uncertain significance (VUS) (21%). Although twin and family studies estimate the overall heritability of autism spectrum disorder (ASD) to range from 40% to 91% [18], with a heritability on the liability scale of 0.924 [19,20], large-scale genomic cohort studies demonstrate that a clear monogenic or syndromic etiology can be identified in only 8% to 16% of clinically confirmed ASD cases [21,22].

In patients presenting with isolated ASD, without comorbid intellectual disability, motor delay, or dysmorphic features, the diagnostic yield of WES is considerably lower, consistent with the complex genetic architecture of ASD. Importantly, a negative WES result does not exclude an underlying monogenic etiology, as non-coding variants, repeat expansions, low-level mosaicism, small or complex structural variants, CNVs below the detection threshold, and variants in technically challenging regions may remain undetected. Genetic factors, encompassing de novo mutations, rare variants (MAF ≤ 0.1%), and common variants of modest effect, account for 20% to 25% of ASD cases [19]. These findings are consistent with those of previous research conducted in Hispanic populations, which indicates that the presence of autism in conjunction with neurodevelopmental delay or intellectual disability enhances the diagnostic efficacy of whole-exome sequencing (WES) [22]. Consequently, our findings suggest that WES diagnostic yield is higher in individuals with ASD accompanied by additional neurodevelopmental or syndromic features, while genetic testing decisions should be guided by the overall clinical context and the technical scope of the testing approach.

The positivity rate is consistent with global ASD trends. In fact, positivity rate varies considerably among populations of diverse ancestral and geographic origins; for example, rates of 27.6% and 11.1% have been documented in Bedouin and Jewish cohorts, respectively, whereas studies in Bulgarian and Chinese populations reported yields of 8.3% and 10% [23,24,25]. Genomic data remain critically scarce in Latin America, particularly in low- and middle-income countries (LMICs) such as Colombia. Based on the searches conducted, this is the first study to provide data on diagnostic yield in real-world clinical practice, obtained from an unselected cohort of patients with ASD from the region. Therefore, these findings lay the groundwork for bridging the genomic knowledge gap and set a precedent for the use of precision medicine tailored to the unique genetic architecture of Hispanic populations, within the framework of an accessible healthcare model.

An additional finding of particular interest was the identification of pathogenic and likely pathogenic variants in genes not currently confirmed to be linked to ASD susceptibility. Many of these genes have traditionally been associated with developmental and epileptic encephalopathies, channelopathies, inherited metabolic disorders, or multisystem syndromes, highlighting the marked genetic heterogeneity and broad pleiotropy that characterize neurodevelopmental disorders. Far from being isolated findings, these results indicate that autism spectrum manifestations may be part of a wide range of neurogenetic conditions, reinforcing growing evidence that ASD shares biological mechanisms with other neurodevelopmental disorders [9,26].

The identification of 73 pathogenic and likely pathogenic variants across 54 distinct genes highlights the marked locus heterogeneity underlying ASD in this Hispanic cohort. Notably, over a fifth of these variants were novel, expanding the known mutational spectrum of neurodevelopmental disorders and underscoring the value of sequencing underrepresented populations. Furthermore, trio-based analysis demonstrated a strong enrichment of de novo heterozygous variants, reinforcing the predominant role of high-penetrance, autosomal dominant monogenic factors in the etiology of severe neurodevelopmental phenotypes.

The genes identified in our cohort can be broadly grouped into three main functional categories. The first includes genes involved in neuronal development, synaptic transmission, and intracellular signaling (e.g., *CACNA1E*, *DNM1*, *GRIN2B*, *MAPK8IP3*, *MAST3*, and *MN1*); although primarily associated with developmental and epileptic encephalopathies, their functions converge on core processes such as neuronal differentiation, circuit formation, and synaptic plasticity. A second category comprises genes regulating neuronal excitability, ion transport, and membrane potential (e.g., *KCNMA1*, *SCN9A*, and *SLC12A2*), strongly supporting the excitation-inhibition (E/I) imbalance hypothesis in ASD pathophysiology [27]. Disruptions in these ion channels can impair network synchronization, calcium homeostasis, and GABAergic signaling during critical neurodevelopmental windows. Finally, a third category includes genes linked to hereditary metabolic disorders and complex neurogenetic syndromes (such as *AMT*, *CBL*, *CPT2*, *NF1*, *PANK2*, *PTPN11*, *PUF60*, *RPS6KA3*, and *SDHA*), highlighting that autistic traits may often represent secondary manifestations of broader metabolic or multisystemic defects [28,29,30].

A clinically relevant finding of our study was the association between phenotypic complexity and molecular diagnostic yield. Patients with a positive molecular diagnosis exhibited a significantly higher phenotypic burden—reflected in a greater number of HPO terms per individual—suggesting that the accumulation of neurodevelopmental, neurological, and syndromic manifestations increases the likelihood of identifying a pathogenic or likely pathogenic variant through exome sequencing. This observation is consistent with previous studies demonstrating that the diagnostic yield of exome sequencing increases substantially in patients with complex phenotypes, particularly when ASD is accompanied by intellectual disability, developmental delay, epilepsy, congenital anomalies, or dysmorphic features [21,22].

Among all the phenotypic characteristics evaluated, Global Developmental Delay (GDD) proved to be the strongest predictor of a positive molecular diagnosis and was the only trait that remained statistically significant after correction for multiple testing. Patients with GDD were approximately three times more likely to harbor a pathogenic or likely pathogenic variant compared to those who did not exhibit this clinical feature. This finding is biologically plausible, as GDD frequently reflects an alteration in fundamental neurodevelopmental processes—such as neuronal differentiation, transcriptional regulation, chromatin remodeling, and synaptic function—mechanisms widely implicated in ASD and other neurodevelopmental disorders [9,26].

Other clinical features, such as microcephaly, macrocephaly, seizures, hypotonia, short stature, and craniofacial anomalies, showed nominal associations with the molecular diagnosis. Although these associations did not remain significant after correction for the false discovery rate, they remain clinically informative, as many of these features are characteristic of syndromic forms of ASD and frequently define subgroups with a higher pre-test probability of a monogenic etiology [31]. The high positivity rate observed in patients with microcephaly and macrocephaly was particularly noteworthy. Abnormal head circumference is a clinically relevant feature, as it is frequently associated with monogenic neurodevelopmental disorders affecting neuronal proliferation, brain growth, and the epigenetic regulation of development [32,33]. Furthermore, the observed association with seizures and hypotonia reinforces the growing evidence of a biological overlap between ASD, developmental and epileptic encephalopathies, and other complex neurogenetic disorders, many of which share alterations in neuronal network formation and excitability [34,35]. Accordingly, the results of this study indicate that heritable phenotypes such as macrocephaly, as well as structural features identified via MRI, and the presence of seizures or electroencephalographic abnormalities, have the potential to contribute to a more precise definition of autism subtypes.

Despite the clinical and molecular diversity of the identified genes, functional enrichment analyses revealed a convergence toward shared biological mechanisms. Reactome analysis showed significant enrichment in pathways related to the neuronal system, chemical synaptic transmission, axon guidance, NTRK receptor signaling, and L1CAM interactions. These pathways play essential roles in neuronal connectivity, synaptic plasticity, and the establishment of neural circuits during development, and are widely recognized as central mechanisms in the pathogenesis of ASD [30,35]. Complementarily, HPO enrichment analysis revealed an overrepresentation of phenotypes related to epilepsy, electroencephalographic abnormalities, and neurodevelopmental dysfunction, while Gene Ontology analyses highlighted processes associated with neuronal excitability, ion transport, and transmembrane signaling.

## 4. Materials and Methods

### 4.1. Study Design and Population

This retrospective, real-world cohort study included all consecutive patients with a clinical diagnosis of ASD referred for genomic testing at the Specialized Laboratory of Clínica Colsanitas in Colombia from 2022 to 2025. Participants were not selected a priori by the research team. Patients were eligible if both genomic data and standardized phenotypic information were available. This inclusive strategy was designed to capture the full clinical and genetic heterogeneity of ASD while decreasing selection bias. The final cohort comprised 916 individuals.

### 4.2. Sample Processing and Analysis

WES was performed in the Specialized Laboratory of Clínica Colsanitas according to the following protocol: Sample processing was performed using the Illumina DNA Prep with Exome 2.5 Enrichment (Illumina, San Diego, CA, USA) and Twist Human Exome 2.0 Plus Exome (Twist Biosciences, South San Francisco, CA, USA). For the paired-end pre-capture library procedure, genomic DNA was fragmented and ligated to multiplexed PE adapters. Adapter-linked DNA was amplified by PCR using sequencing barcode primers (indexes). Subsequently, the pre-capture library was enriched by hybridizing with biotin-labeled probes in solution. For sequencing, post-capture library DNA was subjected to sequence analysis on an NGS sequencing platform, Illumina Novaseq6000 (Illumina, San Diego, CA, USA) or MGI G-400 (MGI Tech Co., San Jose, CA, USA). The quality control metrics typically achieved were: ≥94% of the target base covered at ≥20×, with a mean coverage of the target bases ≥100×.

Variants were classified according to ACMG guidelines and subsequent recommendations by Clingen [36,37]; pathogenic, likely pathogenic, and VUS variants were reported. The report generally included copy number variants (CNVs) that affect three or more exons. However, due to the resolution and limitations of the technique, confirmation of these findings by a CNV-dedicated detection method was recommended. The secondary and tertiary analysis of the obtained sequences was conducted using Varsome Clinical software v13, using GRCh37/Hg19 and GRCh38/Hg38 as the reference genome. Quality metrics, classification, and relationship to patient phenotype were used for variant filtering by the bioinformatics curator team. For this purpose, all HPO terms describing the patient’s phenotype were extracted. A panel of medical geneticists reviewed all analyses to select variants for reporting.

Based on previous studies and existing databases, we selected candidate genes associated with autism and related neurodevelopmental conditions. To identify genes strongly associated with autism or other severe neurodevelopmental disorders, we examined their presence in one of the following databases: SFARI Gene (Categories S, 1–3), SPARK Gene, or the Developmental Brain Disorder Gene Database (DBDGD) (https://dbd.geisingeradmi.org/ accessed on 15 June 2026). Additionally, we included genes with less robust evidence linking them to autism, intellectual disability, or co-occurring conditions such as epilepsy using the autismKB database.

### 4.3. Phenotypic Characterization and Semantic Similarity Analysis

Phenotypic information was encoded using HPO terminology. Across the cohort, 807 unique HPO terms were identified. The terms Autism (HP:0000717) and Autistic behavior (HP:0000729) were excluded from downstream analyses because they were uniformly represented within the cohort and therefore lacked discriminatory value. Patients harboring pathogenic or likely pathogenic variants in prioritized ASD-associated genes were considered molecularly solved. Within this subgroup, 186 unique HPO terms were identified.

To reduce phenotypic dimensionality, a multi-step filtering strategy was applied. First, HPO terms consistently reported in ASD and neurodevelopmental disorder studies were retained. Second, terms lacking disease or gene annotations in OMIM or Orphanet were excluded. Finally, singleton terms (present in only one patient) were removed to reduce noise from non-recurrent observations.

Semantic similarity analysis was performed using Lin’s metric implemented in the HPO3 library. Information content values were derived from OMIM phenotype–disease annotations. Multiple similarity thresholds were evaluated, and a cutoff of 0.5 was selected to maximize phenotypic coverage while preserving cluster specificity.

Terms with semantic similarity scores ≥ 0.5 were grouped into phenotypic clusters. For terms with multiple ancestral relationships, the ancestor yielding the highest similarity score was selected as the representative phenotype. Cluster composition was subsequently reviewed by a clinical geneticist to ensure biological plausibility and remove residual redundancy.

### 4.4. Protein–Protein Interaction Network and Functional Enrichment Analysis

Genes harboring pathogenic or likely pathogenic variants were analyzed using STRING version 12.0. Protein–protein interaction (PPI) networks were generated using a high-confidence threshold (score ≥ 0.70).

Functional enrichment analyses were performed to identify overrepresented Gene Ontology (GO) categories, including Biological Processes, Molecular Functions, Cellular Components, and Protein Classes. Additional pathway enrichment analyses were performed using Reactome and Human Phenotype Ontology annotations to explore the biological convergence of candidate genes.

### 4.5. Statistical Analysis

Continuous variables were summarized as medians and interquartile ranges (IQR), whereas categorical variables were reported as frequencies and percentages. Phenotypic burden was defined as the total number of unique HPO terms recorded per individual and was compared between molecularly solved and unsolved patients using the Wilcoxon rank-sum test. To identify phenotypic predictors of molecular diagnosis, association analyses were performed for the 39 representative HPO features obtained after semantic similarity clustering. Diagnostic yield, odds ratios (ORs), and 95% confidence intervals (95% CIs) were calculated using Fisher’s exact test or chi-square test as appropriate. To account for multiple testing, *p*-values were adjusted using the Benjamini–Hochberg false discovery rate (FDR) procedure. Statistical significance was defined as both nominal *p* < 0.05 and FDR-adjusted *p* < 0.05. All analyses were conducted in R (version 4.x).

### 4.6. Ethical Considerations

This study was approved by the Research Ethics Committee of the Fundación Universitaria Sanitas (CEIFUS code 1419-21, date of approval: 2 July 2021). Informed consent was obtained for the use of samples from participating patients in the study. The study was conducted in accordance with local regulations and the Declaration of Helsinki.

## 5. Conclusions

This study provides the first comprehensive characterization of the genomic landscape of autism spectrum disorder in an unselected Colombian cohort, achieving a diagnostic yield comparable to other non-European populations. Our findings demonstrate that phenotypic complexity—specifically Global Developmental Delay—is the strongest predictor of identifying a pathogenic or likely pathogenic variant via whole-exome sequencing, underscoring the clinical utility of standardized phenotyping using the Human Phenotype Ontology for patient stratification in low-resource and underrepresented settings.

Furthermore, the identification of pathogenic variants in genes traditionally associated with epileptic encephalopathies, channelopathies, and metabolic syndromes illustrates the extensive pleiotropy and mechanistic overlap underlying neurodevelopmental disorders. Despite this clinical heterogeneity, functional analyses confirmed a biological convergence onto shared pathways regulating synaptic transmission, neuronal excitability, and axonal guidance. Overall, this work expands the genomic knowledge of ASD in Latin America and highlights the transformative potential of precision genomic medicine to refine diagnosis, improve clinical management, and narrow global disparities in neurogenetics.

## Figures and Tables

**Table 1:** Classification and segregation of pathogenic and likely pathogenic (P/LP) variants.

Gene	Classification	Transcript	Variant	Segregation
*ABCC8*	P	NM_000352.6	c.3748C>T (p.Arg1250*)	U
*AMT*	LP	NM_000481.4	c.958C>T (p.Arg320Cys)	U
*ANK2*	LP *	NM_001148.6	c.1417C>T (p.Arg473*)	I
*ARID1B*	LP *	NM_017519.2	c.3430_3432del (p.Gln1144del)	U
	P	NM_020732.3	c.2162G>A (p.Gly721Asp)	DN
*BCL11A*	LP	NM_022893.4	c.544G>A (p.Ala182Thr)	DN
*CACNA1E*	LP	NM_001205293.1	c.5365-2A>G	DN
*CACNA1F*	LP *	NM_001256789.3	c.4864C>T (p.Gln1622*)	I
*CAMK2A*	LP	NM_015981.4	c.74C>T (p.Ser25Leu)	DN
*CBL*	LP *	NM_005188.4	c.1103A>G (p.Tyr368Cys)	DN
*CDK8*	P	NM_001260.2	c.185C>T (p.Ser62Leu)	U
*CHD2*	LP	NM_001271.4	c.2822A>T (p.Gln941Leu)	DN
	P	NM_001271.4	c.4173dup (p.Gln1392Thrfs*17)	DN
*CHD8*	P	NM_020920.4	c.3322dup (p.Ile1108Asnfs*7)	DN
	P	NM_001170629.2	c.4987_5003del (p.Val1663Glnfs*59)	DN
*CPT2*	P *	NM_000098.2	c.338C>T (p.Ser113Leu)	I
*CREBBP*	LP	NM_004380.3	c.5141T>C (p.Val1714Ala)	U
	LP	NM_004380.3	c.5312G>A (p.Ser1771Asn)	DN
	P	NM_004380.3	c.6307C>T (p.Gln2103*)	DN
	P	NM_004380.3	c.22G>T (p.Gly8*)	DN
*CTNNB1*	P	NM_001904.5	c.999C>G (p.Tyr333*)	U
*CUL3*	P	NM_003590.5	c.1349_1350del (p.Ser450*)	DN
	P *	NM_003590.5	c.1559del (p.Pro520Glnfs*21)	DN
	P	NM_003590.5	c.764C>G (p.Ser255*)	DN
*DICER1*	LP *	NM_030621.4	c.455del (p.Cys152Serfs*5)	I
*DNM1*	LP *	NM_004408.4	c.1751A>T (p.His584Leu)	DN
*EBF3*	P	NM_001375380.1	c.1429_1441del (p.Thr477Profs*10)	I
*EP300*	P	NM_001429.4	c.4783T>G (p.Phe1595Val)	DN
*FOXP1*	P	NM_032682.6	c.1541G>A (p.Arg514His)	DN
*FBXW11*	LP	NM_001378976.1	c.760T>C (p.Trp254Arg)	U
*GRIN2B*	LP	NM_000834.5	c.3912C>G (p.Tyr1304*)	U
*IQSEC2*	LP	NM_001111125.3	c.2983C>T (p.Arg995Trp)	U
*KCNMA1*	LP	NM_001161352.1	c.2095A>T (p.Lys699*)	I
*KDM6A*	LP	NM_001291415.2	c.3366-1G>A	U
*KMT2C*	LP	NM_170606.3	c.8308_8312del (p.Lys2770Hisfs*3)	I
*LARS2*	P *	NM_015340.4	c.1420del (p.Leu474Trpfs*6)	DN
*MAPK8IP3*	LP	NM_001318852.1	c.3410del (p.Gly1137Alafs*36)	I
*MAST3*	P	NM_015016.2	c.1528G>A (p.Gly510Ser)	DN
*MECP2*	P	NM_004992.3	c.1200_1243del (p.Pro401_Pro402delins*)	DN
	LP	NM_001110792.2	c.433C>T (p.Arg145Cys)	DN
	P	NM_001110792.2	c.473C>T (p.Thr158Met)	I
*MN1*	P	NM_002430.3	c.3883C>T (p.Arg1295*)	U
*MYO6*	LP	NM_004999.4	c.3496C>T (p.Arg1166*)	U
*NAA15*	P	NM_057175.5	c.2214del (p.Met738Ilefs*18)	DN
*NBEA*	LP	NM_001385012.1	c.8362+1G>A	U
*NF1*	LP *	NM_001042492.2	c.2056A>T (p.Lys686*)	U
*PANK2*	LP *	NM_153638.3	c.638G>A (p.Trp213*)	U
*PTEN*	LP	NM_000314.6	c.521A>G (p.Tyr174Cys)	DN
	P	NM_000314.8	c.445C>T (p.Gln149*)	U
*PKD1*	P	NM_001009944.3	c.4796dup (p.Tyr1599*)	I
*POLR3B*	LP	NM_018082.6	c.3273G>A (p.Trp1091*)	I
*PTPN11*	LP	NM_002834.5	c.1282G>A (p.Val428Met)	DN
*PUF60*	LP *	NM_014281.5	c.1334C>T (p.Thr445Ile)	DN
*QRICH1*	P	NM_198880.2	c.136del (p.Gln46Serfs*200)	DN
*RPE65*	P	NM_000329.3	c.370C>T (p.Arg124*)	I
*RPS6KA3*	LP *	NM_004586.3	c.383C>T (p.Pro128Leu)	DN
*RYR1*	LP	NM_000540.3	c.4071del (p.Gly1359Alafs*39)	I
*SATB2*	P	NM_001172509.2	c.1285C>T (p.Arg429*)	DN
*SCN2A*	LP	NM_001040142.1	c.1456_1457del (p.Ser486Phefs*6)	DN
	LP	NM_001040142.2	c.641C>A (p.Ser214*)	I
	LP	NM_001040142.2	c.4694G>A (p.Trp1565*)	U
	P	NM_001040142.1	c.4446+2T>C	U
*SCN9A*	LP *	NM_001365536.1	c.2454_2458del (p.Leu818Phefs*38)	I
*SDHA*	LP	NM_004168.4	c.1064+2T>A	U
*SHANK3*	P	NM_001372044.2	c.2451+1G>A	DN
	P	NM_001372044.2	c.352dup (p.Leu118Profs*28)	DN
*SLC12A2*	LP	NM_001046.3	c.2222_2225dup (p.Leu743Serfs*47)	U
*SPEN*	P	NM_015001.4	c.5485_5486insTTTGAAC (p.Gln1829Leufs*2)	DN
*STXBP1*	P	NM_001032221.3	c.903-1G>C	DN
*SYNGAP1*	P	NM_006772.3	c.1216_1218delins (p.Tyr406Asnfs*4)	DN
	LP	NM_006772.3	c.1735C>T (p.Arg579*)	U
*TAOK1*	LP	NM_020791.4	c.1489_1492del (p.Asp497Lysfs*42)	DN
	LP	NM_020791.4	c.1721dupA (p.Ser575Glufs*28)	I/DN
*TCF20*	P	NM_005650.4	c.2573_2574dup (p.Ser859Valfs*9)	DN
*TCF4*	P	NM_001083962.1	c.1733G>A (p.Arg578His)	I
*TRIO*	P	NM_007118.4	c.3232C>T (p.Arg1078Trp)	DN
	P	NM_007118.4	c.3233G>A (p.Arg1078Gln)	DN
*TNXB*	LP	NM_001365276.2	c.11599del (p.Gln3867Argfs*65)	I
*TUBB2A*	LP *	NM_001069.3	c.55A>G (p.Lys19Glu)	DN
*WDR26*	LP *	NM_025160.7	c.1523G>A (p.Arg508Gln)	DN
*ZMIZ1*	LP	NM_020338.3	c.1413+4A>G	DN

DN: de novo, I: inherited, U: unknown. P: pathogenic; LP: likely pathogenic. Previously unreported variants are designated by an asterisk (*).

**Table 2:** Characterization of the CNVs identified.

Age (Years)	Sex	CNV Type	Cytogenetic Band	Size (Mb)	Genomic Coordinates (GRCh37/hg19)	Copy Number	ACMG Classification
15	Female	Duplication	17p11.2	3.5766	17:16640847-20217403	3	P
8	Male	Deletion	10q26.13-26.3	7.4400	10:125980146-133420180	1	P
4	Female	Deletion	4q35.2	1.9906	4:187074741-189065298	1	P
14	Female	Deletion	22q13.33	0.0876	22:51133160-51220796	1	P
6	Male	Duplication	16p13.11	0.6788	16:14983129-15661936	3	LP
10	Male	Deletion	22q13.31q13.33	5.5842	22:45636613-51220796	1	P
7	Female	Deletion	3p14.2	4.9128	3:61547944-66460744	1	P
7	Male	Deletion	7q22.1	5.1841	7:98445679-103629803	1	LP
7	Male	Deletion	15q12	0.0014	15:27017519-27018871	0	LP
12	Male	Deletion	22q13.33	0.0064	22:51137075-51143524	1	LP

## Data Availability

Supporting Information is available on request due to ethical issues.

## Abbreviations

The following abbreviations are used in this manuscript:

EPS	Entidad Promotora de Salud (in Spanish)
ASD	Autism spectrum disorder

## Appendix A

**Table A1:** Gene-level ASD evidence for genes harboring pathogenic or likely pathogenic variants identified in the study cohort.

Gene	Variant	ACMG Class	Zygosity	Inheritance	Associated Disorder(s)	ASD-Related Evidence	Study Interpretation
*PUF60*	NM_014281.5; c.1334C>T; p.Thr445Ile	LP	HET	AD	Verheij syndrome	Zhao JJ et al. [38]; SFARI 3S (Suggestive Evidence, Syndromic)	Candidate ASD-associated gene
*SCN9A*	NM_001365536.1; c.2454_2458del; p.(Leu818PhefsTer38)	LP	HET	AD/AR, phenotype-dependent	SCN9A-related pain disorders; HSAN type IID	Xiong J et al. [39]; SFARI 2 (Strong Candidate); DBDGD	Candidate ASD-associated gene
*TUBB2A*	NM_001069.3; c.55A>G; p.Lys19Glu	LP	HET	AD (typically de novo)	TUBB2A-related tubulinopathy/complex cortical dysplasia	Schmidt L et al. [40]; SFARI: not listed; DBDGD	Limited ASD-related evidence
*RPE65*	NM_000329.3; c.370C>T; p.(Arg124Ter)	P	HOM	AR (classic retinal dystrophy)	RPE65-related inherited retinal dystrophy/Leber congenital amaurosis	Patowary A et al. [41]; AutismKB	Limited ASD-related evidence
*MN1*	c.3883C>T; p.Arg1295Ter	P	—	AD (typically de novo)	MN1 C-terminal truncation syndrome	Hussain SI et al. [42]	Limited ASD-related evidence
*LARS2*	c.1420del; p.Leu474TrpfsTer6	P	—	AR	Perrault syndrome 2/LARS2-related mitochondrial disease	Blaze J et al. [43]	Limited ASD-related evidence
*DNM1*	c.1751A>T; p.His584Leu	LP	—	AD (usually de novo)	DNM1-related developmental and epileptic encephalopathy	SFARI 3 (Suggestive Evidence)	Candidate ASD-associated gene
*SLC12A2*	c.2222_2225dup; p.(Leu743SerfsTer47)	LP	—	AD (often de novo)	SLC12A2-related neurodevelopmental/cochleovestibular disorder	McNeill A et al. [44]	Limited ASD-related evidence
*PANK2*	NM_153638.3; c.638G>A; p.(Trp213*)	LP	HET	AR	Pantothenate kinase-associated neurodegeneration (PKAN)	Dong S et al. [45]; DBDGD	Limited ASD-related evidence; single HET variant does not establish AR disease
*ABCC8*	NM_000352.4; c.3748C>T; p.Arg1250*	P	HET	AD/AR, mechanism-dependent	ABCC8-related hyperinsulinism and monogenic/neonatal diabetes	Miyake N et al. [46]; DBDGD	Limited ASD-related evidence
*AMT*	NM_000481.4; c.958C>T; p.Arg320Cys	LP	HOM	AR	Glycine encephalopathy 2	Yu TW et al. [47]; SFARI 2 (Strong Candidate)	Candidate ASD-associated gene
*ANK2*	NM_001148.6; c.1417C>T; p.Arg473*	LP	HET	AD	ANK2-related neurodevelopmental disorder; ankyrin-B cardiac arrhythmia	Iossifov I et al. [48]; SFARI 1 (High Confidence); AutismKB; DBDGD	Established ASD-associated gene
*CACNA1F*	NM_001256789.3; c.4864C>T; p.Gln1622*	LP	HEMI	X-linked	CACNA1F-related retinal disorders	SFARI 2 (Strong Candidate); AutismKB	Candidate ASD-associated gene
*CPT2*	NM_000098.2; c.338C>T; p.Ser113Leu	P	HET	AR	Carnitine palmitoyltransferase II deficiency	Balasar Ö et al. [49]; SFARI 2 (Strong Candidate); AutismKB	Candidate ASD-associated gene; variant-level causality requires caution
*EBF3*	NM_001375380.1; c.1429_1441del; p.Thr477Profs*10	P	HET	AD (usually de novo)	Hypotonia, ataxia, and delayed development syndrome (HADDS)	Tanaka AJ et al. [50]; SFARI 1S (High Confidence, Syndromic)	Established ASD-associated gene
*GRIN2B*	NM_000834.5; c.3912C>G; p.Tyr1304*	LP	HET	AD (usually de novo)	GRIN2B-related neurodevelopmental disorder/DEE	Yoo HJ et al. [51]; SFARI 1 (High Confidence); AutismKB	Established ASD-associated gene
*KCNMA1*	NM_001161352.1; c.2095A>T; p.Lys699*	LP	HET	AD/AR, phenotype-dependent	KCNMA1-related channelopathy (developmental delay, seizures and/or paroxysmal dyskinesia)	Laumonnier F et al. [52]; SFARI 2 (Strong Candidate); AutismKB	Candidate ASD-associated gene
*MAPK8IP3*	NM_001318852.1; c.3410del; p.Gly1137Alafs*36	LP	HET	AD (usually de novo)	MAPK8IP3-related neurodevelopmental disorder	Yuen RK et al. [53]; SFARI 2S (Strong Candidate, Syndromic); AutismKB	Candidate ASD-associated gene
*MAST3*	NM_015016.2; c.1528G>A; p.Gly510Ser	P	HET	AD (usually de novo)	MAST3-related developmental and epileptic encephalopathy	Yuen RK et al. [53]; SFARI 3 (Suggestive Evidence)	Candidate ASD-associated gene
*MYO6*	NM_004999.4; c.3496C>T; p.Arg1166*	LP	HET	AD/AR, phenotype-dependent	MYO6-related nonsyndromic hearing loss	AutismKB	Limited ASD-related evidence
*PANK2*	NM_153638.3; c.1561G>A; p.Gly521Arg	LP	HET	AR	Pantothenate kinase-associated neurodegeneration (PKAN)	AutismKB	Limited ASD-related evidence; single HET variant does not establish AR disease
*PKD1*	c.4796dup; p.Tyr1599*	P	HET	AD	Polycystic kidney disease 1	Yuen RK et al. [53]; SFARI 3 (Suggestive Evidence); AutismKB	Candidate ASD-associated gene
*POLR3B*	c.3273G>A; p.Trp1091*	LP	HET	AR; dominant NDD mechanism also reported	POLR3B-related hypomyelinating leukodystrophy/neurodevelopmental disorder	AutismKB	Limited ASD-related evidence; mechanism/segregation should be considered
*PTPN11*	c.1282G>A; p.Val428Met	LP	HET	AD	Noonan syndrome/Noonan syndrome with multiple lentigines	Tartaglia M et al. [54]; SFARI 1S (High Confidence, Syndromic); AutismKB	Established ASD-associated gene
*RPS6KA3*	c.383C>T; p.Pro128Leu	LP	HET	X-linked	Coffin–Lowry syndrome/RPS6KA3-related intellectual developmental disorder	O’Roak BJ et al. [55]; SFARI 2S (Strong Candidate, Syndromic); AutismKB	Candidate ASD-associated gene
*RYR1*	c.4071del; p.Gly1359Alafs*39	LP	HET	AD/AR, phenotype-dependent	RYR1-related disorders: malignant hyperthermia susceptibility and congenital myopathies	AutismKB	Limited ASD-related evidence; primarily associated with another clinical phenotype
*SDHA*	c.1064+2T>A	LP	HET	Mechanism-dependent	SDHA-related mitochondrial disease/tumor predisposition/rare NDD presentations	AutismKB	Limited ASD-related evidence
*TNXB*	c.11599del; p.Gln3867Argfs*65	LP	HET	AR (classical-like EDS)	TNXB-related classical-like Ehlers–Danlos syndrome	AutismKB	Limited ASD-related evidence; primarily associated with another clinical phenotype
*CACNA1E*	c.5365-2A>G	LP	HET	AD (usually de novo)	CACNA1E-related developmental and epileptic encephalopathy	O’Roak BJ et al. [55]; SFARI 1 (High Confidence); AutismKB	Established ASD-associated gene
*NF1*	c.2056A>T; p.Lys686*	LP	HET	AD	Neurofibromatosis type 1/NF1-related disorders	SFARI 1S (High Confidence, Syndromic); AutismKB; Marui T et al. [56]	Established ASD-associated gene
*DICER1*	c.455del; p.Cys152Serfs*5	LP	HET	AD	DICER1 tumor-predisposition syndrome	AutismKB	Limited ASD-related evidence; primarily associated with another clinical phenotype

Abbreviations: P, pathogenic; LP, likely pathogenic; HET, heterozygous; HOM, homozygous; HEMI, hemizygous; AD, autosomal dominant; AR, autosomal recessive; XLD, X-linked dominant; XLR, X-linked recessive.

**Table A2:** Phenotypic features associated with a positive molecular diagnosis.

Phenotypic Feature	N	Positive Cases	Yield (%)	OR (95% CI)	*p*-Value	FDR-Adjusted *p*
Global Developmental Delay	121	25	20.7	3.07 (1.76–5.24)	4.34 × 10^−5^	0.0344
Microcephaly	9	5	55.6	12.5 (2.63–64.31)	6.42 × 10^−4^	0.2550
Neurodevelopmental Abnormality	19	7	36.8	5.94 (1.92–16.9)	1.08 × 10^−3^	0.2850
Macrocephaly	40	10	25.0	3.45 (1.45–7.6)	2.77 × 10^−3^	0.4850
Short Stature	28	8	28.6	4.09 (1.51–10.08)	3.05 × 10^−3^	0.4850
Seizure	66	13	19.7	2.57 (1.23–5.05)	7.39 × 10^−3^	0.7320
Abnormal Facial Shape	26	7	26.9	3.72 (1.28–9.6)	8.05 × 10^−3^	0.7320
Skeletal Muscle Atrophy	2	2	100.0	Inf (1.8–Inf)	8.93 × 10^−3^	0.7320
Hypotonia	31	7	22.6	2.93 (1.03–7.29)	2.17 × 10^−2^	0.7320
Abnormal Affect	3	2	66.7	19.34 (1–1141.03)	2.51 × 10^−2^	0.7320
Receptive Language Delay	3	2	66.7	19.34 (1–1141.03)	2.51 × 10^−2^	0.7320
Weight Loss	3	2	66.7	19.34 (1–1141.03)	2.51 × 10^−2^	0.7320
Anteverted Nares	9	3	33.3	4.88 (0.78–23.37)	4.56 × 10^−2^	0.7320
Depressed Nasal Bridge	9	3	33.3	4.88 (0.78–23.37)	4.56 × 10^−2^	0.7320
Hypertelorism	9	3	33.3	4.88 (0.78–23.37)	4.56 × 10^−2^	0.7320
Precocious Puberty	9	3	33.3	4.88 (0.78–23.37)	4.56 × 10^−2^	0.7320
Neonatal Asphyxia	4	2	50.0	9.68 (0.69–135.22)	4.71 × 10^−2^	0.7320
Poor Speech	4	2	50.0	9.68 (0.69–135.22)	4.71 × 10^−2^	0.7320
Scoliosis	29	6	20.7	2.59 (0.84–6.8)	4.89 × 10^−2^	0.7320
